# Colistin resistance mutations in *phoQ* can sensitize *Klebsiella pneumoniae* to IgM-mediated complement killing

**DOI:** 10.1038/s41598-023-39613-5

**Published:** 2023-08-03

**Authors:** Sjors P. A. van der Lans, Manon Janet-Maitre, Frerich M. Masson, Kimberly A. Walker, Dennis J. Doorduijn, Axel B. Janssen, Willem van Schaik, Ina Attrée, Suzan H. M. Rooijakkers, Bart W. Bardoel

**Affiliations:** 1 Department of Medical Microbiology, University Medical Center Utrecht, Utrecht University, Utrecht, The Netherlands; 2 Bacterial Pathogenesis and Cellular Responses Group, UMR5075, Institute of Structural Biology, University Grenoble Alpes, Grenoble, France; 3 Department of Molecular Microbiology, Washington University School of Medicine, St. Louis, Missouri USA; 4 Department of Microbiology and Immunology, University of North Carolina, Chapel Hill, North Carolina USA; 5 Department of Fundamental Microbiology, University of Lausanne, Lausanne, Switzerland; 6 Institute of Microbiology and Infection, College of Medical and Dental Sciences, University of Birmingham, Birmingham, UK

**Keywords:** Bacterial pathogenesis, Bacterial immune evasion, Antibodies, Complement cascade, Immune evasion, Antimicrobial responses, Bacterial infection, Infection

## Abstract

Due to multi-drug resistance, physicians increasingly use the last-resort antibiotic colistin to treat infections with the Gram-negative bacterium *Klebsiella pneumoniae.* Unfortunately, *K. pneumoniae* can also develop colistin resistance. Interestingly, colistin resistance has dual effects on bacterial clearance by the immune system. While it increases resistance to antimicrobial peptides, colistin resistance has been reported to sensitize certain bacteria for killing by human serum. Here we investigate the mechanisms underlying this increased serum sensitivity, focusing on human complement which kills Gram-negatives via membrane attack complex (MAC) pores. Using in vitro evolved colistin resistant strains and a fluorescent MAC-mediated permeabilization assay, we showed that two of the three tested colistin resistant strains, Kp209_CSTR and Kp257_CSTR, were sensitized to MAC. Transcriptomic and mechanistic analyses focusing on Kp209_CSTR revealed that a mutation in the *phoQ* gene locked PhoQ in an active state, making Kp209_CSTR colistin resistant and MAC sensitive. Detailed immunological assays showed that complement activation on Kp209_CSTR in human serum required specific IgM antibodies that bound Kp209_CSTR but did not recognize the wild-type strain. Together, our results show that developing colistin resistance affected recognition of Kp209_CSTR and its killing by the immune system.

## Introduction

Infections with antibiotic-resistant bacteria form a serious threat for public health. Among the most concerning bacteria is the Gram-negative bacterium *Klebsiella pneumoniae*, which caused nearly 1 million antibiotic resistance associated deaths in 2019 alone^1^. In the past decades, the number of antibiotic resistant *K. pneumoniae* has risen dramatically for several classes of antibiotic^2–4^. Propagation of antibiotic resistance often occurs in nosocomial settings, where antibiotic resistant strains are transferred between patients and antibiotic resistance genes can spread between *K. pneumoniae* lineages^1,5^. To treat infections caused by antibiotic resistant *K. pneumoniae*, clinicians increasingly need to use last resort antibiotics such as colistin. Colistin, a membrane-targeting antibiotic, is attracted to the Gram-negative cell envelope via electrostatic interactions^6^. The Gram-negative cell envelope consists of a thin peptidoglycan cell wall in between an outer and an inner membrane, with negatively charged lipopolysaccharides (LPS) being present in the outer leaflet of the outer membrane. The positively charged colistin is attracted to these negative charges in the cell envelope, causing colistin to insert into the membranes. This will subsequently destabilise and disrupt both the outer and inner membrane, ultimately leading to cell death^7^.

Previously, it was shown that development of colistin resistance in Gram-negative bacteria has a negative impact on bacterial fitness and virulence in vivo^8–10^. Intriguingly, various different effects have been observed in vitro. While antimicrobial peptides and lysozyme were less effective against colistin resistant strains^9,11^, killing of *K. pneumoniae* and *Escherichia coli *in vitro by human serum was more efficient in colistin resistant strains^8,12^. Although human serum contains several antimicrobial components, the main antimicrobial effector in serum is the complement system, a protein network that can directly kill Gram-negative bacteria via the formation of the membrane attack complex (MAC). MAC formation is initiated after bacteria are recognized by the complement system, which leads to sequential deposition of complement proteins on the bacterial surface. This ultimately results in the formation of the MAC, a multi-protein complex that forms a pore in the outer membrane, leading to inner membrane destabilization and bacterial killing^13^.

In this study, we aimed to investigate how colistin resistance affects bacterial sensitivity to human complement. Using a sensitive fluorescent MAC assay and a panel of in vitro evolved colistin resistant *K. pneumoniae* strains^9^, we identified two isogenic strain pairs, Kp209/Kp209_CSTR and Kp257/Kp257_CSTR, in which the colistin resistant (CSTR) mutant exhibited increased sensitivity to MAC. Transcriptomic and mutational analysis of Kp209_CSTR reveal that a mutation locking PhoQ in an active state made this strain resistant to colistin and sensitive to MAC at the same time. Detailed immunological assays show that bacterium-specific IgMs mediate enhanced MAC sensitivity in the colistin-resistant Kp209_CSTR.

## Results

### Inner membrane damage correlates with MAC-mediated killing of *K. pneumoniae*

**Figure 1:**
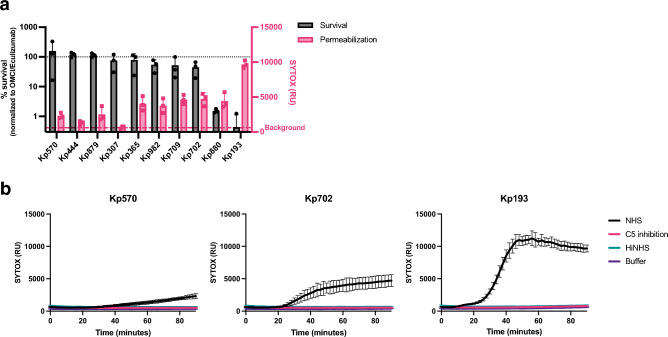
MAC-dependent inner membrane permeabilization correlates with reduced viability of *K. pneumoniae*. (**a**) Survival on plate and inner membrane permeabilization of clinical *K. pneumoniae* isolates after 90-min incubation in 10% normal human serum (NHS) at 37 °C in the presence of 1 µM SYTOX green nucleic acid stain. Survival data was normalized to CFU counts in conditions where C5 conversion was inhibited by addition of 20 µg/ml OMCI and 20 µg/ml Eculizumab. Inner membrane permeabilization (SYTOX fluorescence intensity) was determined in a microplate reader. Red dotted line indicates background permeabilization signal (OMCI + Eculizumab). (**b**) Inner membrane permeabilization of Kp570, Kp702 and Kp193 in the presence of 10% NHS, 10% NHS in which C5 conversion was inhibited by addition of 20 µg/ml OMCI and 20 µg/ml Eculizumab (C5 inhibition), or 10% heat inactivated NHS (HiNHS). Bacteria were incubated at 37 °C in the presence of 1 µM SYTOX green nucleic acid stain, and inner membrane permeabilization (SYTOX fluorescence intensity) was detected every 2 min for 90 min in a microplate reader. (**a**,**b**) Data represent mean ± standard deviation of three independent experiments.

To study the relation between colistin resistance and MAC sensitivity, we first established an in vitro assay system to quantify MAC-mediated killing of *K. pneumoniae* in microplates*.* Previously, we showed that fluorescent DNA dyes can be used as a proxy for MAC-dependent killing of *E. coli*. Specifically, we used Sytox DNA dyes that can only stain Gram-negative bacteria when both the outer and inner membrane are damaged^13^. We observed that MAC-mediated inner membrane damage of *K. pneumoniae* in human serum correlated with bacterial killing on plate^13^. To determine if this assay can be used for *K. pneumoniae* clinical isolates, we analysed the MAC-mediated membrane permeabilization and killing of ten clinical *K. pneumoniae* strains. Bacteria were incubated with 10% normal human serum (NHS) containing both complement and naturally occurring antibodies. Inner membrane permeabilization was monitored by measuring fluorescence over time and compared to bacterial survival that was assessed by counting colony forming unites (CFU) on plate (Fig. 1a,b).

First, we observed that four out of ten tested isolates were resistant to MAC-mediated killing (Kp570, Kp444, Kp879 and Kp307), as these strains showed no decrease in survival (Fig. 1a). No or a minimal increase in inner membrane damage for these four MAC resistant isolates was seen (Fig. 1a,b, Supplementary Fig. 1). Furthermore, the strain that was most sensitive to MAC-mediated killing (Kp193) showed the strongest membrane permeabilization (Fig. 1a). Inner membrane damage started after 20 min in Kp193, after which the signal quickly increased and reached a plateau after 40 min (Fig. 1b). Five strains showed a slow increase in membrane permeabilization (Kp365, Kp982, Kp709, Kp702 and Kp880) (Fig. 1a,b, Supplementary Fig. 1). Most of these strains showed only a minor reduction in survival. No inner membrane permeabilization was observed when MAC formation was blocked by serum heat-inactivation or addition of a C5 cleavage inhibitor. Taken together, these data indicate that for *K. pneumoniae*, MAC activity can be quantified by measuring inner membrane damage via Sytox green.

### Developing colistin resistance sensitizes *K. pneumoniae* Kp209 and Kp257 to MAC-mediated killing

Since previous studies demonstrated that colistin resistance can affect serum sensitivity of certain *K. pneumoniae* isolates^8,11,12^, we wondered whether there was a role for MAC in this process. First, we used our fluorescent MAC activity assay to investigate the influence of colistin resistance on MAC-mediated killing of *K. pneumoniae.* To allow a direct comparison, we used three isogenic *K. pneumoniae* strain pairs that were previously selected during an in vitro evolution experiment, in which three colistin sensitive *K. pneumoniae* strains (Kp209, Kp257 and Kp040) were exposed to increasing concentrations of colistin to evolve colistin-resistant (CSTR) strains (Kp209_CSTR, Kp257_CSTR and Kp040_CSTR)^9^. Colistin resistance in Kp209_CSTR and Kp257_CSTR was linked to mutations in the *phoQ* gene (WAQRN-97-C and G385S, respectively). Kp040_CSTR had a IS5 insertion in the promoter region of *crrAB and crrC,* and a point mutation in the promoter region of *ecpR* or *phnC*^9^. Mutations in these genes have been associated with colistin resistance, which was attributed to a reduced negative charged of the outer membrane due to altered lipid A modifications^9,14–17^.

**Figure 2:**
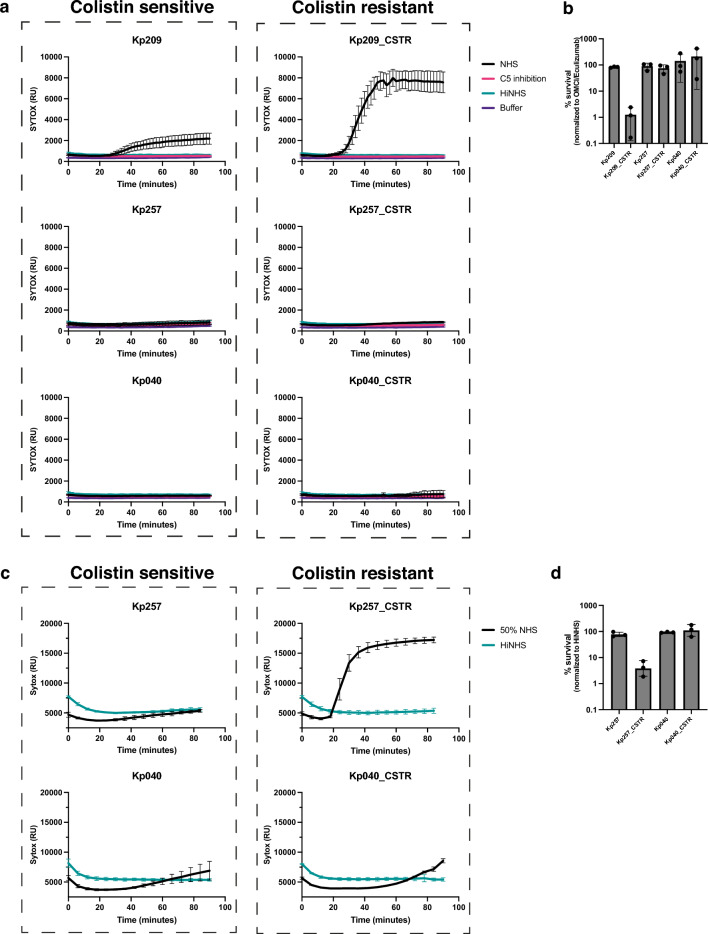
Colistin resistant Kp209_CSTR has been sensitized to MAC-mediated killing. (**a**) Inner membrane permeabilization of Kp209, Kp257 and Kp040, and their colistin resistant derived strains Kp209_CSTR, Kp257_CSTR and Kp040_CSTR in the presences of 10% NHS, 10% NHS in which C5 conversion was inhibited by addition of 20 µg/ml OMCI and 20 µg/ml Eculizumab (C5 inhibition), or 10% heat inactivated NHS (HiNHS). Bacteria were incubated at 37 °C in the presence of 1 µM SYTOX green nucleic acid stain, and inner membrane permeabilization (SYTOX fluorescence intensity) was detected every 2 min for 90 min in a microplate reader. (**b**) Survival on plate of Kp209, Kp257 and Kp040, and their colistin resistant derived strains Kp209_CSTR, Kp257_CSTR, and Kp040_CSTR after 90 min incubation in 10% NHS at 37 °C. Survival data is normalized to CFU counts in conditions where the terminal complement pathway was blocked by inhibiting C5 conversion (20 µg/ml OMCI and 20 µg/ml Eculizumab). (**c**) Inner membrane permeabilization of Kp257, Kp257_CSTR, Kp040 and Kp040_CSTR in the presence of 50% NHS, or 50% HiNHS. Bacteria were incubated at 37 °C in the presence of 1 µM SYTOX green nucleic acid stain, and inner membrane permeabilization (SYTOX fluorescence intensity) was detected every 6 min for 90 min in a microplate reader. (**d**) Survival on plate of Kp257, Kp257_CSTR, Kp040 and Kp040_CSTR after 90 min incubation in 50% NHS at 37 °C. Survival data was normalized to CFU counts in 50% HiNHS. (**a**–**d**) Data represent mean ± standard deviation of three independent experiments.

In this study, we exposed these three isogenic strain pairs to NHS and quantified MAC-mediated membrane damage. While acquisition of colistin resistance increased the MAC-dependent permeabilization of Kp209_CSTR in 10% NHS, no effect was observed for Kp257_CSTR or Kp040_CSTR (Fig. 2a). In concordance, colony enumeration experiments confirmed that Kp209_CSTR was killed more effectively by NHS than Kp209 (Fig. 2b). Membrane damage of Kp209_CSTR was caused by MAC, as blocking complement activation via a C5 conversion inhibitor or heat inactivation of NHS prevents membrane permeabilization. Similar results were obtained using NHS composed of sera of a different pool of donors, and with sera from individual donors for Kp209 and Kp209_CSTR (Supplementary Fig. 2). As Kp257_CSTR has a mutation in the same gene (*phoQ*) as Kp209_CSTR, but was not sensitive to 10% NHS, we decided to increase the concentration of NHS. When measuring membrane permeabilization and serum survival in 50% NHS, we observed that Kp257_CSTR was permeabilized and killed, whereas Kp257 was not, indicating that acquisition of colistin resistance also increased serum sensitivity of Kp257_CSTR (Fig. 2c,d). No permeabilization or killing was observed for Kp040 and Kp040_CSTR in 50% NHS (Fig. 2c,d)^8,12^. Taken together these data confirm that developing colistin resistance can lead to increased MAC-sensitivity, although this does not happen for every *K. pneumoniae* strain.

### Overactivation of PhoQ sensitizes Kp209_CSTR to MAC-mediated membrane permeabilization

**Figure 3:**
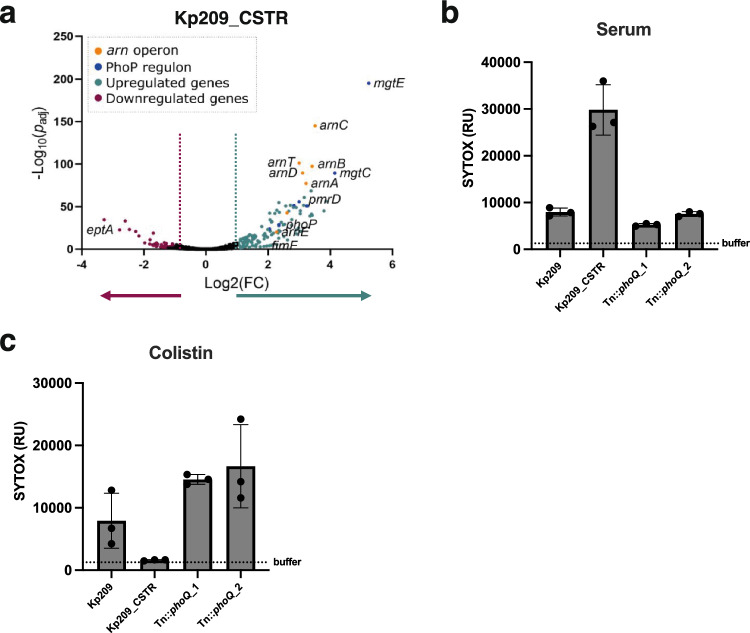
Colistin resistant Kp209_CSTR has been sensitized to MAC-mediated killing due to enhanced PhoQ activation. (**a**) RNA was isolated from log phase Kp209 and Kp209_CSTR, and their transcriptomes were analysed. The volcano plot depicts differential expressed genes in Kp209_CSTR compared to Kp209. In Kp209_CSTR 124 genes were upregulated and 60 downregulated. (**b**,**c**) Kp209_CSTR transposon (Tn) library was exposed to 16% NHS, followed by re-exposed to 32% NHS, and surviving colonies were selected. Two unique Tn mutants had a transposon insertion in *phoQ* (Tn::*phoQ*). (**b**) Inner membrane permeabilization of Kp209, Kp209_CSTR, and Kp209_CSTR Tn::*phoQ* mutants in the presence of 10% NHS. (**c**) Inner membrane permeabilization of Kp209, Kp209_CSTR, and Kp209_CSTR Tn::*phoQ* mutants in the presence of 1 µg/ml colistin. (**b**,**c**) Bacteria were incubated at 37 °C in the presence of 1 µM SYTOX green nucleic acid stain, and inner membrane permeabilization (SYTOX fluorescence intensity) was detected after 60 min in a microplate reader. Data represent mean ± standard deviation of three independent experiments.

Genetic comparison of Kp209 and Kp209_CSTR revealed that Kp209_CSTR exclusively contains mutations in the *phoQ* gene^12^, a sensor histidine kinase that is part of the PhoPQ two-component regulatory system^22^. More specifically, the *phoQ* gene of Kp209_CSTR contains a WAQRN-97-C deletion located in the sensory domain of PhoQ^12,43^. In *Salmonella*, it was shown that disruption of tryptophan residue at position 97 (W-97) locks PhoQ in an active state^44,45^. To investigate if PhoQ in Kp209_CSTR is locked in an active state, we compared the gene expression of Kp209 and Kp209_CSTR, which indicated increased transcriptional activation of the PhoPQ regulon in Kp209_CSTR (Fig. 3a). 124 genes were significantly upregulated (Log2(FC) > 1, Padj < 0.05) in Kp209_CSTR, whereas 60 genes were downregulated (Log2(FC) < − 1, Padj < 0.05) (Supplementary Table 1). Of the upregulated genes, many are known to be dependent on PhoQ activity, such as genes coding for proteins involved in magnesium transport and LPS modifications^22,25^. To determine if PhoPQ regulated genes are also upregulated in Kp257_CSTR, which has a G385S mutation in the histidine kinase domain of PhoQ, we measured the relative expression of *arnD* and *mgtE* in Kp257 and Kp257_CSTR, as these genes were among the strongest upregulated genes in Kp209_CSTR. We observed an increased relative expression of 13.8-fold and 7.8-fold for *arnD* and *mgtE*, respectively, in Kp257_CSTR compared to Kp257 (Supplementary Fig. 3). This indicated that the *phoQ* colistin resistance mutation in Kp257_CSTR also promotes expression of PhoPQ regulated genes, similar to what we observed for Kp209_CSTR.

To directly prove that PhoQ plays a role in MAC sensitivity of Kp209_CSTR, we generated a transposon (Tn) library of Kp209_CSTR and exposed it to human serum. The library was first challenged with 16% NHS, followed by re-exposure of surviving bacteria to 32% NHS. Out of the surviving bacteria, eight colonies were picked and the *phoQ* gene was analysed. This revealed that three out of the eight Tn mutants had a transposon insertion in *phoQ* (Tn::*phoQ*). Two of the three Tn::*phoQ* mutants were clonally related as they had the same transposon at an identical insertion site. We analysed the MAC-mediated membrane permeabilization of the two unique Tn::*phoQ* mutants, which showed reduced membrane permeabilization compared to Kp209_CSTR (Fig. 3b), indicating that loss of *phoQ* leads to decreased MAC sensitivity. We also assessed colistin sensitivity of the Tn::*phoQ* mutants. As colistin disrupts the bacterial cell envelop to kill bacteria, the membrane permeabilization assay could be used to compare colistin sensitivity between Kp209, Kp209_CSTR and the Tn::*phoQ* mutants (Fig. 3c). Kp209_CSTR was not permeabilized by colistin, whereas the colistin sensitive Kp209 was. Both Kp209_CSTR Tn::*phoQ* mutants were permeabilized by colistin, indicating that loss of PhoQ function leads to increased colistin sensitivity of this strain. Taken together these data indicate that enhanced PhoQ activity, as a result of colistin resistance mutations, led to an increased MAC-sensitivity in Kp209_CSTR.

### Capsule production is reduced in Kp209_CSTR

Enhanced PhoPQ activity due to colistin resistance mutations has been linked to decreased capsule production in *K. pneumoniae*^11,12^. Therefore, we tested capsule production of Kp209_CSTR, and found that it was reduced compared to Kp209 (Supplementary Fig. 4). This reduction was the result of the constitutively active PhoQ, as disrupting this gene by transposon insertion restored capsule production to level of Kp209 (Supplementary Fig. 4). The LPS O-antigen, the other major extracellular polysaccharide of *K. pneumoniae* was not different in length and abundance between Kp209 and Kp209_CSTR (Supplementary Fig. 4).

### Classical pathway activation is crucial for killing of Kp209_CSTR

**Figure 4:**
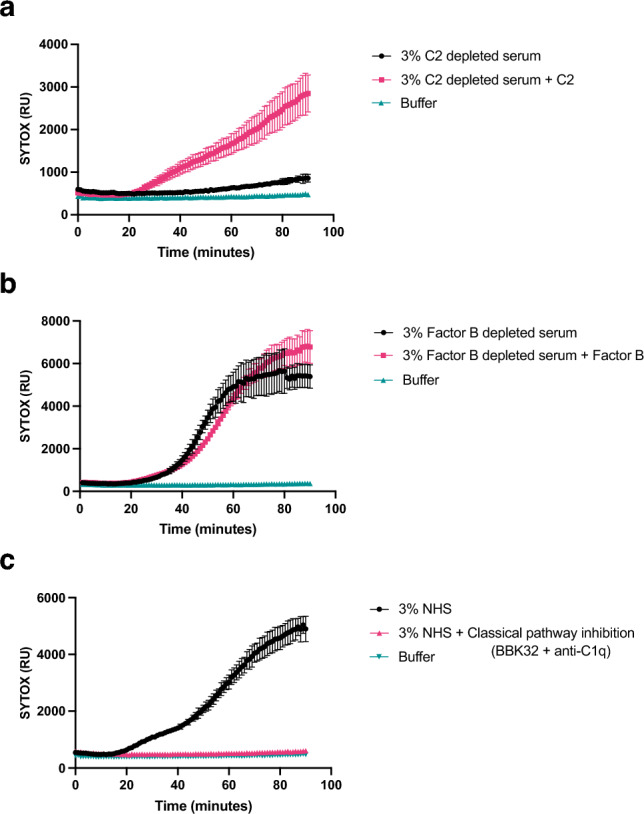
MAC-dependent inner membrane permeabilization of Kp209_CSTR is dependent on the classical pathway. (**a**) Inner membrane permeabilization of Kp209_CSTR in the presence of 3% C2 depleted serum or C2 depleted serum reconstituted with C2 to physiological concentrations (0.6 µg/ml in 3% serum). (**b**) Inner membrane permeabilization of Kp209_CSTR in the presence of 3% fB depleted serum or fB depleted serum reconstituted with fB to the physiological concentration (6 µg/ml in 3%). (**c**) Inner membrane permeabilization of Kp209_CSTR in the absence or presence of the classical pathway inhibitors anti-hu-C1q and BBK32 (both 10 µg/ml) in 3% NHS. (**a**–**c**) Bacteria were incubated at 37 °C in the presence of 1 µM SYTOX green nucleic acid stain, and inner membrane permeabilization (SYTOX fluorescence intensity) was detected every 2 min for 90 min in a microplate reader. Data represent mean ± standard deviation of three independent experiments.

To study the mechanism that caused increased MAC-mediated killing of Kp209_CSTR, we first analysed which complement pathway was responsible for MAC-dependent permeabilization. The complement system can be activated via three distinct pathways (classical, lectin and alternative pathway) which are activated via different mechanisms, but all lead to the formation of MAC on Gram-negative bacteria. Both the classical and the lectin pathway depend on complement protein C2, therefore we used C2 depleted serum to determine the involvement of these pathways. We observed that depletion of C2 resulted in the complete loss of membrane permeabilization of Kp209_CSTR (Fig. 4a). This effect was restored by adding back C2 to physiological levels, which indicated that C2 was crucial for complement-mediated membrane damage of Kp209_CSTR. Similarly, factor B (fB) depleted serum was used to determine the role of the alternative pathway. Depletion of fB did not influence inner membrane permeabilization of Kp209_CSTR (Fig. 4b). These data suggest that killing of Kp209_CSTR is primarily driven by the classical and/or lectin pathway. To further distinguish between the classical and lectin pathway, we used inhibitors to specifically block the first steps of classical pathway activation. The initiation of the classical pathway starts when IgG or IgM antibodies bind the bacterial surface. Upon binding, these antibodies can recruit the large C1 complex, which is specific to the classical pathway. C1 consists of the recognition protein C1q and associated proteases C1r and C1s. To block C1 dependent complement activation, we simultaneously added both a monoclonal antibody that directly prevents C1q association to surface-bound antibodies, and the C1r inhibitor BBK32^18–20^. Addition of these inhibitors to NHS prevented inner membrane damage of Kp209_CSTR (Fig. 4c). In summary, these findings indicate that MAC-mediated membrane permeabilization of Kp209_CSTR in these conditions primarily depends on the classical pathway.

### IgM specific for Kp209_CSTR is responsible for MAC-mediated membrane permeabilization of Kp209_CSTR

**Figure 5:**
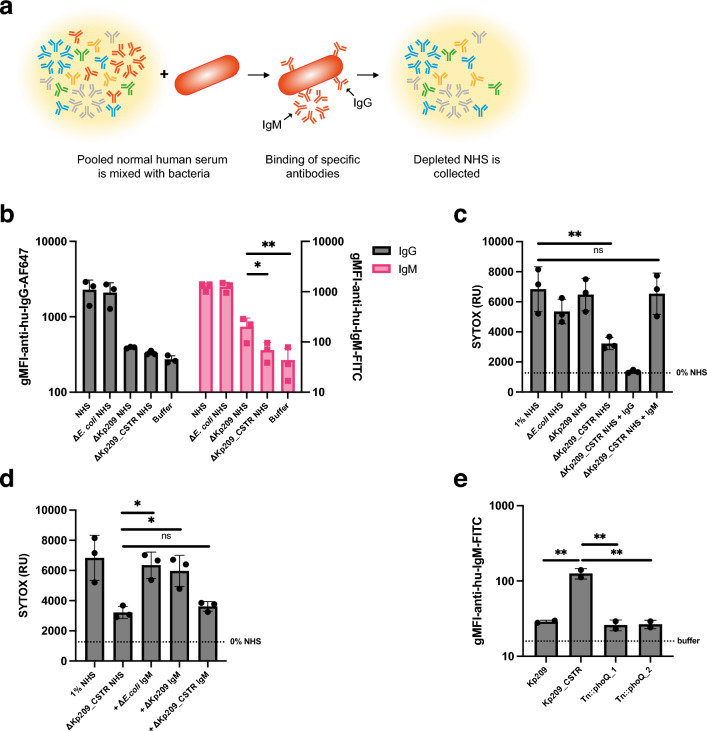
NHS contains Kp209_CSTR specific IgM that is vital for MAC-dependent inner membrane permeabilization. (**a**) Schematic representation of method to deplete bacterium specific antibodies from NHS. NHS was mixed with bacteria and incubated for 10 min at 4 °C to allow specific antibodies to bind. Bacteria were pelleted and discarded, and the depleted NHS was collected. Three rounds of depletion were performed. Image was created using Adobe Illustrator v27.7. (**b**) IgG and IgM binding to Kp209_CSTR in NHS, NHS depleted with *E. coli* MG1655, Kp209 or Kp209_CSTR (Δ*E. coli* NHS, ΔKp209 NHS, and ΔKp209_CSTR NHS, respectively. (**c**) Inner membrane permeabilization of Kp209_CSTR in the presence of 1% NHS or NHS depleted using *E. coli* MG1655, Kp209 or Kp209_CSTR (Δ*E. coli* NHS, ΔKp209 NHS, and ΔKp209_CSTR NHS, respectively). ΔKp209_CSTR NHS was supplemented with physiological concentrations of IgG (+ IgG; 125 µg/ml in 1% NHS) or IgM (+ IgM; 15 µg/ml in 1% NHS) isolated from NHS. (**d**) Inner membrane permeabilization of Kp209_CSTR in the presence of 1% NHS depleted using Kp209_CSTR (ΔKp209_CSTR NHS), supplemented with physiological concentrations of IgM isolated from NHS (15 µg/ml in 1% NHS), depleted using *E. coli* MG1655, Kp209 or Kp209_CSTR (+ Δ*E. coli* IgM, + ΔKp209 IgM, and + ΔKp209_CSTR IgM, respectively). (**e**) IgM binding to Kp209, Kp209_CSTR, and Kp209_CSTR Tn::*phoQ* mutants in 10% NHS depleted using Kp209. Data represent mean ± standard deviation of 2 independent experiments. (**b**,**e**) IgG and IgM binding was performed in 0.3% and 10% (depleted) NHS, respectively. Binding was detected using anti-hu-IgG-AF647 or anti-hu-IgM-FITC by flow cytometry. Flow cytometry data are represented by geometric mean fluorescent intensity (gMFI) values of bacterial populations. (**c**,**d**) Bacteria were incubated at 37 °C in the presence of 1 µM SYTOX green nucleic acid stain, and inner membrane permeabilization (SYTOX fluorescence intensity) was detected after 60 min. (**b**–**d**) Data represent mean ± standard deviation of three independent experiments. (**b**–**e**) Statistical analysis was performed using a paired one-way ANOVA with a Tukey’s multiple comparisons test on SYTOX fluorescence intensity (**c**,**e**) or Log_10_-transformed gMFI data (**b**,**d**). Significance is shown as *p ≤ 0.05, **p ≤ 0.005, ***p ≤ 0.005, ****p ≤ 0.0005.

The sensitivity of Kp209_CSTR to the classical complement pathway suggests a role for IgG or IgM in the increased MAC-dependent membrane permeabilization in NHS. We hypothesized that there might be increased binding of IgG or IgM to Kp209_CSTR in NHS, but incubation of Kp209 and Kp209_CSTR with NHS revealed that there was no major difference in total IgG or IgM binding to these strains (Supplementary Fig. 5). As IgG and IgM are polyclonal in NHS, we analysed if NHS contains antibodies that specifically recognize Kp209_CSTR, but not Kp209. A relative low concentration of Kp209_CSTR-specific antibodies could explain why we did not observe a difference in the total antibody binding between Kp209 and Kp209_CSTR. To investigate this hypothesis, we depleted antibodies from NHS using whole bacteria (Fig. 5a). NHS was incubated with Kp209, Kp209_CSTR, or *E. coli* used as a control, at 4 °C to allow antibody binding without activating complement, followed by the removal of bacteria to collect NHS deprived of bacterium-specific antibodies. Three rounds of depletion where sufficient to remove most strain-specific antibodies (Fig. 5b and Supplementary Fig. 5). Depleting NHS with Kp209 removed all IgG binding to Kp209_CSTR (Fig. 5b), and vice versa (Supplementary Fig. 5), indicating that both strains were bound by the same IgGs in NHS. Depletion with Kp209_CSTR abolished IgM binding to Kp209 (Supplementary Fig. 5), showing that all the IgM that bound Kp209 also recognized Kp209_CSTR. However, although depletion with Kp209 strongly reduced IgM binding to Kp209_CSTR, considerable binding was still detectable, implying that NHS contains Kp209_CSTR-specific IgM (Fig. 5b). We validated that antibodies were specifically removed from NHS by *K. pneumoniae* depletion*,* as they did not affect antibody binding to *E. coli* (Supplementary Fig. 5).

The finding that NHS contained Kp209_CSTR-specific IgM raised the question whether IgM played a role in classical pathway activation. Therefore, we incubated Kp209_CSTR in Kp209_CSTR-depleted NHS and observed that membrane permeabilization was reduced (Fig. 5c). Membrane permeabilization of *E. coli* was not altered in Kp209_CSTR-depleted NHS, indicating that the membrane permeabilizing potential was not affected by the depletion (Supplementary Fig. 5). To test if IgM was required for membrane permeabilization of Kp209_CSTR, we supplemented Kp209_CSTR-depleted NHS with polyclonal IgG or IgM purified from NHS and monitored bacterial membrane permeabilization. Addition of polyclonal IgM fully restored membrane permeabilization on Kp209_CSTR, whereas polyclonal IgG did not (Fig. 5c). Similar results were obtained when Kp209_CSTR-depleted NHS was supplemented with isolated IgM of individual donors (Supplementary Fig. 5). NHS depletion using Kp209 or *E. coli* did not affect complement-mediated membrane damage on Kp209_CSTR, indicating that components required for complement activation on Kp209_CSTR were still present (Fig. 5c). This suggested that KP209_CSTR-specific IgM was responsible for complement-mediated membrane damage.

To verify that Kp209_CSTR-specific IgM was responsible for classical pathway activation, we used the antibody depletion technique to deplete polyclonal IgM isolated form NHS. Similar to NHS, depletion of polyclonal IgM with Kp209 reduced the IgM binding to Kp209_CSTR but again residual binding was observed, confirming the presence of Kp209_CSTR-specific IgM (Supplementary Fig. 5)*.* Depletion of polyclonal IgM using Kp209 or Kp209_CSTR did not affect binding to *E. coli* (Supplementary Fig. 5). Next, we supplemented NHS depleted with Kp209_CSTR with the different preparations of IgM to study the effect on membrane permeabilization of Kp209_CSTR. IgM depleted with Kp209 or *E. coli* was able to restore membrane permeabilization on Kp209_CSTR, in contrast to IgM depleted with Kp209_CSTR (Fig. 5d). This confirmed that Kp209_CSTR-specific IgM is crucial for antibody driven complement activation on Kp209_CSTR. As Kp209_CSTR has a constitutively active PhoQ, we were curious if Kp209_CSTR-specific IgM binding would be lost after *phoQ* disruption. To this end, we incubated the Kp209_CSTR Tn::*phoQ* mutants with Kp209-depleted NHS and measured IgM binding (Fig. 5e). IgM binding to the Tn::*phoQ* mutants was comparable to the binding signal observed for Kp209. This indicates that PhoQ activity is required for Kp209_CSTR-specific IgM binding. In summary, we found that human serum contains IgM specific for Kp209_CSTR that induces MAC-mediated inner membrane damage.

## Discussion

Membrane attack complex (MAC) insertion into the outer membrane is important for the direct killing of Gram-negative bacteria by the complement system. In the past, resistance against the membrane-targeting antibiotic colistin has been shown to influence sensitivity to serum in Gram-negative bacteria^8,12^. Here, we aimed to study the effect of developing colistin resistance on MAC-mediated killing of *K. pneumoniae*. Of the three colistin resistant strains tested, both Kp209_CSTR and Kp257_CSTR became more MAC-sensitive. The *phoQ* mutations responsible for colistin resistance in these strains enhanced expression of PhoQ regulated genes, indicating that a more active form of PhoQ leads to increased MAC-sensitivity*. *Vice versa, inactivation of *phoQ* in Kp209_CSTR decreased MAC-dependent membrane permeabilization and colistin resistance. Finally, we demonstrated that MAC-mediated membrane permeabilization of Kp209_CSTR in NHS was activated by IgM that specifically recognized Kp209_CSTR.

The finding that MAC-mediated membrane permeabilization of Kp209_CSTR is driven by Kp209_CSTR-specific IgM suggests that an epitope for IgM becomes exposed on Kp209_CSTR due to the mutation in *phoQ*. This epitope might be shielded by capsular polysaccharides on the wild-type Kp209, and becomes available on Kp209_CSTR due to the reduction in capsule production. A role for a thicker capsule in shielding bacterial outer membrane and associated antigens from recognition by antibodies was demonstrated in previous studies. For example, chemical repression of capsule production by *K. pneumoniae* was demonstrated to lead to enhanced monoclonal antibody binding to the LPS^21,22^. Similarly, reduced capsule production due to genetic modifications changed the ability of the capsule to shield outer membrane proteins of *K. pneumoniae*^23,24^.

NHS and IgM was isolated from healthy donors with no described prior exposure to *K. pneumoniae*. The question remains why NHS contained IgM that specifically recognized Kp209_CSTR. One possibility is that the IgM that targeted Kp209_CSTR was a natural antibody, which are defined as germline-encoded antibodies expressed without any known direct antigenic stimulus^25^. Natural antibodies targeting bacterial antigens have been found in mice, and are assumed to exist in humans as well^26^. On the other hand, *K. pneumoniae* is a human commensal residing in the gut microbiota and nasopharynx, and estimations of gastrointestinal carriage in the western world range from 5 to 45%^27,28^. It is therefore probable that the serum IgM binding to Kp209_CSTR is part of a specific antibody response against antigens originating from commensal *K. pneumoniae.* This is supported by reports that healthy individuals produce both IgM and IgG antibodies recognizing *K. pneumoniae*^29–33^. Furthermore, the antibodies of healthy donors recognizing *K. pneumoniae* antigens belonging to the IgG, IgA and IgM isotypes can be highly affinity-maturated^34,35^.

Only a minority of IgMs that bound to the surface of Kp209*_*CSTR were essential for inducing MAC-mediated membrane permeabilization. A large part of the IgM that bound Kp209_CSTR also bound to Kp209, but only the Kp209_CSTR-specific IgM was crucial for triggering inner membrane permeabilization. This indicates that the IgM target is an important determinant for MAC-dependent membrane damaging effects. This difference might relate to the location of the target in relation to the outer membrane. For *K. pneumoniae* it has been reported that MAC deposition needs to occur close to the outer membrane to be bactericidal, and that localization of MAC far from the outer membrane prevents bacterial lysis^36^. This would suggest that the target of the bactericidal IgM would be located in close proximity to the outer membrane. However, it remains unclear which epitope the bactericidal IgM targets. Due to the large number of alternatively expressed genes, it was not possible to pinpoint a target using the transcriptomics data for Kp209_CSTR. We observed increased expression for several outer membrane proteins, fimbriae, porin, and efflux pump genes, as well as for genes involved in LPS modification, indicating that both surface proteins and LPS remain potential targets^21,37,38^. Further studies might elucidate which surface structures are targeted, but this will be complicated due the polyclonal nature of IgM. For now, it remains unclear whether the Kp209_CSTR-specific IgM binding that specifically recognizes Kp209_CSTR is monoclonal and bind to one antigen, or a polyclonal mixture that targets multiple different epitopes^21,37,38^.

We found that colistin resistance mutations in *phoQ* had an opposite effect on the MAC sensitivity of Kp209_CSTR and Kp257_CSTR. This might be explained by the different mode of action between colistin and MAC. Although colistin and MAC both interact with and destabilize bacterial membranes, their mechanisms are critically different. Colistin is a small amphipathic molecule attracted to the cell envelope via electrostatic interactions, where it inserts into and destabilizes the bacterial membranes^6^. Antibody-induced MAC deposition is initiated after specific recognition of bacterial surface structures by antibodies. This activates of the classical complement cascade, resulting in sequential deposition of complement components on the bacterial surface. Only in the final stages of the complement cascade, when C5 convertases are being deposited, membrane piercing MAC pores can be formed^13^. As colistin and MAC both target bacterial membranes, but act via different mechanisms, becoming resistant to colistin could have a different effect on MAC sensitivity, which was the case for Kp209_CSTR and Kp257_CSTR.

In contrast to Kp209_CSTR and Kp257_CSTR, colistin resistance did not enhance MAC sensitivity of Kp040_CSTR under the tested conditions. This indicates that colistin resistance can occur without influencing MAC sensitivity. There have been two previous reports on *K. pneumoniae* and *E. coli* where it was shown that colistin resistant mutants were more serum sensitive compared to wild-type strains^8,12^*.* For *E. coli*, increased serum sensitivity was found in two of the three tested strains, indicating that also for *E. coli* colistin resistance not always leads to increases serum sensitivity^8^. The previous study on *K. pneumoniae* showed that all three tested strains became more serum sensitive^12^. These were all ST23, whereas strains tested in our study belong to different sequence types. This suggests that in different genetic backgrounds serum sensitivity can be affected differently after acquiring colistin resistance. Overall, there is a connection between colistin resistance and increased MAC-sensitivity, but variation between different strains remains to be explained.

Transcriptome analysis revealed that the WAQRN-97-C deletion in the sensory domain of PhoQ led to a constitutively active form of PhoQ in Kp209_CSTR^9,39^. The tryptophan residue at position 97 (W-97) is essential to the sensory function of PhoQ in *S. enterica*, and its loss results in a to a constitutively active PhoQ^40,41^. In concordance with our own results, a deletion in the PhoQ sensory domain in one of previously reported *K. pneumoniae* ST23 led to upregulation of PhoPQ regulated genes, indicating that PhoQ was more active^12^. We found that PhoQ activity influences capsule production, which might play a role in binding of Kp209_CSTR-specific IgM. Transcriptomic analysis revealed that expression of capsule genes was not altered, suggesting that all the capsule producing machinery should be present in Kp209_CSTR. This suggests that capsule production was not affected on a transcriptional level, but at a later stage of capsule synthesis. It has been postulated that addition of 4-amino-4-deoxy-l-arabinose (L-Ara4N) to lipid A, one of the modifications observed for Kp209_CSTR, could influence capsule production^9,42^. The precursor of L-Ara4N, UDP-glucuronic acid, is also required for capsule production^42–44^. Increased L-Ara4N synthesis would limit the availability of UDP-glucuronic acid, thereby reducing capsule production. This is supported by reports that capsule production can be enhanced by mutating LPS synthesis genes in *E. coli*^43^. UDP-glucuronic acid is converted to L-Ara4N by the Arn pathway. In line with the hypothesis, genes of the Arn pathway, which is responsible for conversion of UDP-glucuronic adic into L-Ara4N, were strongly upregulated in Kp209_CSTR. The *arn* operon is under control the PmrAB two-component system, which is activated by PmrD^45^. The expression of *pmrD* is regulated by PhoPQ and was strongly upregulated in Kp209_CSTR as a result of the mutations in *phoQ*^46^.

As the number of antibiotic resistant *K. pneumoniae* strains rises, infections with these bacteria becomes an increasing risk to human health. Studying how the complement system kills *K. pneumoniae* will reveal new insights in the infection biology of *K. pneumoniae*. Understanding the interaction between killing of *K. pneumoniae* by the immune system and antibiotics will help to improve treatment of *K. pneumoniae* infections.

## Methods

### Bacterial strains

*Klebsiella pneumoniae* clinical isolates were collected during routine diagnostics in the medical microbiology department in the University Medical Centre Utrecht, The Netherlands. *Klebsiella pneumoniae* Kp209, Kp257 and Kp040, and their colistin resistant daughter strains were kindly provided by Axel Janssen (University Medical Centre Utrecht, The Netherlands; University of Lausanne, Switzerland^9^). KPPR1S and KPPR1S*ΔwcaJ* were kindly provided by Kimberly Walker (University of North Carolina, USA^47^). For the experiments with *Escherichia coli*, the laboratory strain MG1655 was used.

### Serum preparation and reagents

Normal human serum (NHS) was prepared as described before^13^. In short, blood was drawn from healthy volunteers, allowed to clot, and centrifuged to separate serum from the cellular fraction. Serum of 15–20 donors was pooled and stored at − 80 °C. Heat inactivation (Hi) of NHS was achieved by incubating NHS at 56 °C for 30 min. OMCI was produced and purified as previously described^48^. RPMI (ThermoFisher) supplemented with 0.05% human serum albumin (HSA, Sanquin), further referred to as RPMI buffer, was used in all experiments, unless otherwise stated. Eculizumab was kindly provided by Frank Beurskens, Genmab, Utrecht, The Netherlands. Sera deficient from factor B (fB) and complement component C2 were obtained from Complement Technology. Purified factor B and C2 were produced by U-protein express (Utrecht, The Netherlands). C1r protease inhibitor BBK32 was kindly provided by Brandon Garcia, Greenville, NC, USA. Monoclonal mouse anti-hu-C1q IgG1 4A4B11 was produced in house (ATCC HB-8327)^20,49^.

### Bacterial growth

For all experiments, bacteria were cultured on Lysogeny broth (LB) 1.5% agar plates at 37 °C, unless stated otherwise. Single colonies were picked and cultured overnight in liquid LB medium at 37 °C while shaking. The following day the bacteria were subcultured by diluting the overnight culture 1/100 in fresh medium and grown to OD_600_ = 0.4–0.5 at 37 °C while shaking. Bacteria were washed twice with RPMI buffer by centrifugation at 10,000*g* for 2 min and resuspended to OD_600_ = 0.5 in RPMI buffer. For the Kp209_CSTR Tn mutants, both solid and liquid medium were supplemented with 30 µg/ml kanamycin.

### Bacterial viability assay

Bacteria (OD_600_ = 0.05) were incubated in serum diluted in RPMI buffer. In the conditions where C5 conversion was blocked to prevent the initiation to the terminal complement pathway, 20 µg/ml OMCI + 20 µg/ml Eculizumab was added. After incubation at 37 °C, tenfold serial dilutions in RPMI buffer were prepared and plated. Colony forming units were determined on LB agar plates after culturing the bacteria on plate over night at 37 °C. Survival in NHS was normalized to NHS in which C5 conversion was inhibited ((#NHS/#C5 inhibition) × 100%), or to NHS in which complement was heat inactivated ((#NHS/#HiNHS) × 100%).

### Membrane permeabilization

Bacteria (OD_600_ = 0.05) were incubated in serum or colistin diluted in RPMI buffer in the presence of 1 µM SYTOX Green Nucleic Acid stain (ThermoFisher). Bacteria were incubated at 37 °C under shaking conditions. Fluorescence was determined in a microplate reader (CLARIOstar, Labtech) using an excitation wavelength of 490–14 nm and an emission wavelength of 537–30 nm.

### Antibody deposition

Bacteria (OD_600_ = 0.05) were incubated in HiNHS diluted in RPMI buffer for 30 min at 4 °C under shaking conditions. Bacteria were washed twice by centrifugation with RPMI buffer, resuspended 1 µg/ml goat anti-hu-IgG-AF647 (2040-31, SouthernBiotech) or 2 µg/ml goat anti-hu-IgM-FITC (2020-02, SouthernBiotech) for 30 min at 4 °C while shaking. Bacteria were washed twice with RPMI buffer and fixated in 1.5% paraformaldehyde in PBS for 5 min. Fluorescence was determined via flow cytometry (MACSQuant, Milteny Biotech), acquiring 10,000 events per condition. Flow cytometry data was analysed in FlowJo V.10. Bacteria were gated based on the forward and side scatter, and AF647 and FITC geometric mean fluorescence intensity was determined for the bacterial population.

### Serum depletion with bacterium

Bacteria (OD_600_ = 1.0) were incubated in ice cold NHS (20% in RPMI buffer). After a 10-min incubation on ice while shaking, bacteria were pelleted in a cooled centrifuge (2 min at 10,000*g*) and the bacterium depleted NHS was collected. The depletion steps were preformed trice in total. Antibody depletion was verified via flow cytometry.

### Polyclonal IgG and polyclonal IgM isolation from NHS

IgG and IgM were isolated from NHS as previously described^20^. In short, IgG was isolated using 5 ml HiTrap Protein G High Performance column (GE Healthcare), whereas IgM was isolated using POROS™ CaptureSelect™ IgM Affinity Matrix (ThermoScientific) in a XK column (GE Healtcare) using an ÄKTA FPLC system. After capture antibodies were eluted according to the manufacturer’s instructions. Collected antibodies were dialyzed overnight against PBS at 4 °C, and stored at − 80 °C.

### Transcriptome analysis

#### RNA extraction

Subcultures of Kp209 and Kp209_CSTR were grown to OD_600_ = 0.5–0.6 at 37 °C while shaking. RNA was isolated using the hot phenol–chloroform method. Briefly, bacteria were incubated in hot phenol lysis solution (1% SDS, 2 mM EDTA, 40 mM sodium acetate in acid phenol (Invitrogen #15594-047)) for 45 min at 65 °C. Total RNA was purified by successive steps of phenol–chloroform extraction, followed by an extraction with cold chloroform. After ethanol precipitation, genomic DNA was digested by Turbo DNAse (Invitrogen #AM1907) treatment. Total RNA quantity and quality were assessed using an Agilent Bioanalyzer.

#### RNA-seq libraries construction

Ribosomal RNAs (rRNAs) depletion was performed using Ribominus bacteria 2.0 transcriptome isolation kit (Invitrogen) according to manufacturer’s instructions. Depletion efficacy was verified on an Agilent Bioanalyzer using RNA pico chips and remaining RNAs were concentrated using ethanol precipitation. The Collibri stranded RNA library prep kit for Illumina (Invitrogen) was used to build cDNA libraries for sequencing starting from 15 ng of RNA according to manufacturer’s instructions. cDNA libraries quality and concentration were assessed using an Agilent Bioanalyzer High sensitivity DNA chip. RNA-seq was performed in biological triplicates.

#### Sequencing and data analysis

Sequencing was performed by the Montpellier GenomiX platform (MGX, https://www.mgx.cnrs.fr/) using an Illumina Novaseq instrument. Following quality control, Bowtie2 was used for sequencing reads mapping^50,51^ onto the genome of Kp209 available on the European Nucleotide Archive under accession number PRJEB29521^9^, previously annotated with Prokka. The read count per feature was calculated using Ht-seq count^52^. Finally, differential gene expression between the Kp209 and Kp209_CSTR strains was performed using DESeq2^53^.

### RNA isolation, reverse transcription and real-time PCR

Subcultures of Kp257 and Kp257_CSTR were grown to OD_600_ = 0.5 at 37 °C while shaking. Total RNA was isolated for three biological replicates per strain using the RNeasy Protect Bacteria Kit (74524, QIAGEN) the manufacturer’s protocol, using lysozyme (L6876, Sigma-Aldrich) and proteinase K (19131, QIAGEN) to lyse the bacteria. DNA was digested on column using the RNase-Free DNase set (79254, QIAGEN), and isolated RNA was subsequently treated with RNase-free DNase I (M0303, New England BioLabs) to remove residual genomic DNA according to the manufacturer’s protocols. RNA was converted to cDNA by reverse transcription using the iScript cDNA Synthesis Kit (1708890, Bio-Rad) according to the manufacturer’s protocols. Real-time PCR was performed using 2 × FastStart Universal SYBER Green Master (Rox) (4913850001, Roche), with each primer at 300 nM and 0.5 ng cDNA input. Primers (Supplementary Table 2) for *arnD* and *rpoB* were selected from previous publications^12,54^. Primers for *mgtE* designed using the PrimerQuest tool available on the webserver of Integrated DNA technologies using the coding sequence of *mgtE* of Kp257^9^. Reactions were performed in triplicate on a StepOnePlus Real-Time PCR System (ThermoFisher), activating the FastStart Taq DNA polymerase for 10 min at 95 °C, followed by 40 cycles of denaturation at 95 °C for 15 s, and quantifications at 56 °C for 1 min. Melt-curve analysis was performed from 56 to 95 °C a ramp of 0.5 °C every 5 s to assess primer specificity. Relative expression of *arnD* and *mgtE* compared to *rpoB* was calculated.

### Transposon library and serum exposure

Kp209_CSTR was mutagenized via conjugation using strain WM3064 carrying the pKMW7 vector with a barcoded Tn5 transposon library as described previously^55^. Bacteria with a transposon insertion were selected using LB supplemented with kanamycin and the library was stored (OD_600_ = 1) at − 80 °C. For serum exposure experiments, transposon library was tenfold diluted in LB medium and cultured to mid-log (OD_600_ = 0.6). Bacteria were pelleted and resuspended in RPMI buffer. Washed bacteria (OD_600_ = 0.05) were incubated with RPMI buffer or 16% NHS for 2 h at 37 °C. Samples were diluted tenfold in LB medium and incubated overnight at 37 °C. The overnight culture was diluted 100-fold in fresh LB medium and challenged with 32% NHS for 2 h at 37 °C followed by serial dilutions in PBS and plating on LB agar. Surviving colonies were picked that survived the 32% NHS challenge for further analysis.

### Capsule production analysis

Capsule production was assessed by measuring the uronic acid content as previously described^47^. Briefly, 500 µl stationary phase bacteria grown in LB medium added to 100 µl 1% Zwittergent 3–14 detergent in citric acid (100 mM pH 2.0) and incubated at 50 °C for 20 min. 300 µl supernatant was collected after centrifugation (5 min 16,000*g*), mixed with 1200 µl 100% ethanol, and incubated at 4 °C overnight. After centrifugation (5 min 16,000*g*), the pellet was dissolved in 200 µl distilled water and 1200 µl tetraborate (12.5 mM in sulfuric acid) was mixed in by vortexing. After a 5-min incubation at 100 °C, the samples were cooled and 20 µl 3-hydroxydiphenol (0.15% in 0.5% sodium hydroxide) was added. Absorbance was measured at 520 nm. Uronic acid amounts were calculated from a standard curve prepared with glucuronolactone and normalized to the culture density (OD_600_).

### LPS silver stain

LPS silver stain was performed as previously described^56^. Briefly, single bacterial colonies were scraped of agar plates, and heat-inactivated at 56 °C for 1 h, followed by protein digestion using proteinase K (400 µg/ml) for 90 min at 60 °C. Samples were diluted in 2 × Laemmli buffer with 0.7 M β-mercaptoethanol, ran over a 4–12% BisTris gel and fixed overnight in ethanol (40% v/v) + glacial acetic acid (4% v/v). The gel was oxidised for 5 min in ethanol (40% v/v) + glacial acetic acid (4% v/v) + periodic acid (0.6% m/v) and stained for 15 min in silver nitrate (0.6% m/v in 0.125 M sodium hydroxide + 0.3% ammonium hydroxide v/v). The gel was developed for 7 min in citric acid (0.25% m/v) + formaldehyde (0.2% v/v).

### Data analysis and statistical testing

Unless stated otherwise data collected as three biological replicates and analysed using GraphPad Prism version 9.4.1 (458). Statistical analyses are further specified in the figure legends.

### Ethics statement

Human blood was isolated after informed consent was obtained from all subjects in accordance with the Declaration of Helsinki. Approval was obtained from the medical ethics committee of the UMC Utrecht, The Netherlands.

### Supplementary Information

Supplementary Information.

## Data Availability

The RNA-seq data generated in this study were deposited on the NCBI Gene Expression Omnibus (GEO) and is publicly available under the GSE212413 accession number (https://www.ncbi.nlm.nih.gov/geo/query/acc.cgi?acc=GSE212413). The datasets generated during and/or analysed during the current study are available from the corresponding author on reasonable request.
