# Supplementary material for: Colistin resistance mutations in phoQ can sensitize Klebsiella pneumoniae to IgM-mediated complement killing
Source: Sci Rep. 2023 Aug 3;13:12618. doi: 10.1038/s41598-023-39613-5 (PMC10400624; doi:10.1038/s41598-023-39613-5)
Supplement: Supplementary file 1 — Supplementary Information. [file 41598_2023_39613_MOESM1_ESM.pdf]

Supplementary information

Colistin resistance mutations in *phoQ* can sensitize *Klebsiella pneumoniae* to IgM-mediated complement killing

Sjors P.A. van der Lans[1], Manon Janet-Maitre[2,3], Frerich M. Masson[1], Kimberly A. Walker[4], Dennis J. Doorduijn[1], Axel B. Janssen[1,5], Willem van Schaik[1,6], Ina Attrée[2], Suzan H. M. Rooijakkers[1] and Bart W. Bardoel[1]*

[1]Department of Medical Microbiology, University Medical Center Utrecht, Utrecht University, The Netherlands.
[2]Bacterial Pathogenesis and Cellular Responses group, UMR5075, Institute of Structural Biology, University Grenoble Alpes, France.
[3]Department of Molecular Microbiology, Washington University School of Medicine, St. Louis, Missouri, USA
[4]Department of Microbiology and Immunology, University of North Carolina, USA.
[5]Department of Fundamental Microbiology, University of Lausanne, Switzerland.
[6]Institute of Microbiology and Infection, College of Medical and Dental Sciences, University of Birmingham, United Kingdom.
*Corresponding author: B.W.Bardoel-2@umcutrecht.nl

Supplementary figure SI1

Inner membrane permeabilization of Kp444, Kp879, Kp307, Kp365, Kp982, Kp709 and Kp880 in time. Bacteria were incubated in the presence of 10% NHS, 10% NHS in which C5 conversion was inhibited by addition of 20 µg/ml OMCI and 20 µg/ml Eculizumab (C5 inhibition), or 10% heat inactivated NHS (HiNHS), at 37 °C in the presence of 1 µM SYTOX green nucleic acid stain, and inner membrane permeabilization (SYTOX fluorescence intensity) was detected every 2 minutes for 90 minutes in a microplate reader. Data represent mean ± standard deviation of three independent experiments.

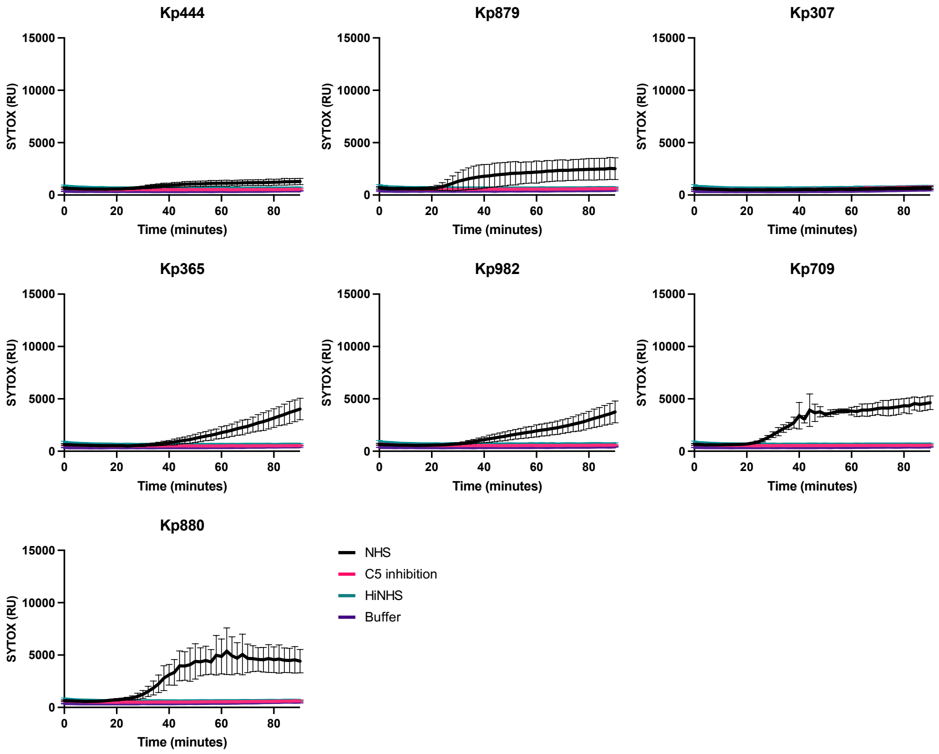

Supplementary figure SI2

(**a**) Inner membrane permeabilization of Kp209 and Kp209_CSTR in the presences of (**a**) 10% NHS composed of sera from different pools of donors, or (**b**) 10% serum from different individual donors. (a&b) Bacteria were incubated at 37 °C in the presence of 1 µM SYTOX green nucleic acid stain, and inner membrane permeabilization (SYTOX fluorescence intensity) was detected every 2 minutes for 90 minutes in a microplate reader. Data represent mean ± standard deviation of three independent experiments.

a

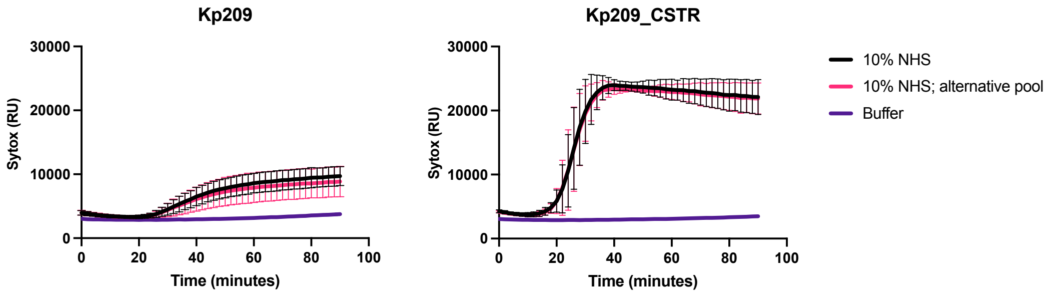

b

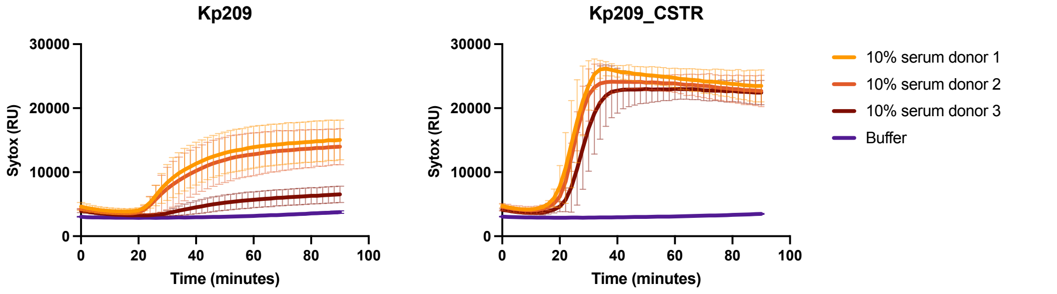

Supplementary figure SI3

RNA was isolated from log phase Kp257 and Kp257_CSTR, and converted to cDNA. The relative expression of the *arnD* and *mgtE* genes compared to the *rpoB* control gene was determined by real-time PCR for both Kp257 and Kp257_CSTR. Real-time PCR was performed in triplicates. Data represent mean ± standard deviation of at least two independent biological replicates.

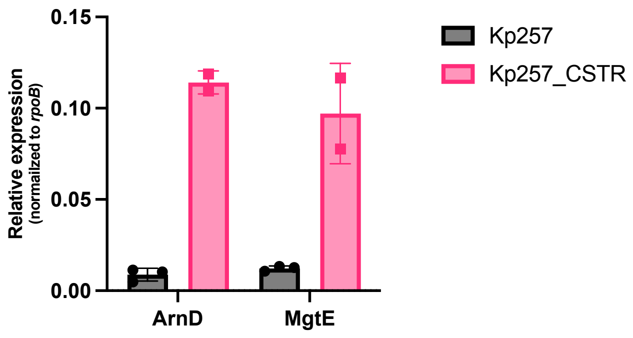

Supplementary figure SI4

(**a**) Capsule production of Kp209 and Kp209_CSTR, and the Kp209_CSTR Tn::*phoQ* mutants was determined by measuring uranic acid content. Absorbance was measured at 520 nm, the uronic acid content calculated using a glucuronolactone standard curve and normalized to the culture density at 600 nm. Data represent mean ± standard deviation of three independent experiments. (F) Statistical analysis was performed using a paired one-way ANOVA with a Tukey's multiple comparisons test. Significance is shown as ****p≤0.0005. (**b**) SDS-PAGE silver stain of Kp209 and Kp209_CSTR LPS. Bacterial lysates were digested with protein K to remove all proteins and loaded on an SDS-PAGE gel. The LPS lipid A could, based on size, be distinguished from the O-antigen, which can have various lengths. Image is a representative of two independent experiments. Image is a representative of two independent experiments. Original gel is presented in supplementary Figure S2c. (c) Original gel of the SDS-PAGE silver stain containing of Kp209 and Kp209_CSTR LPS, indicated with dashed lines.

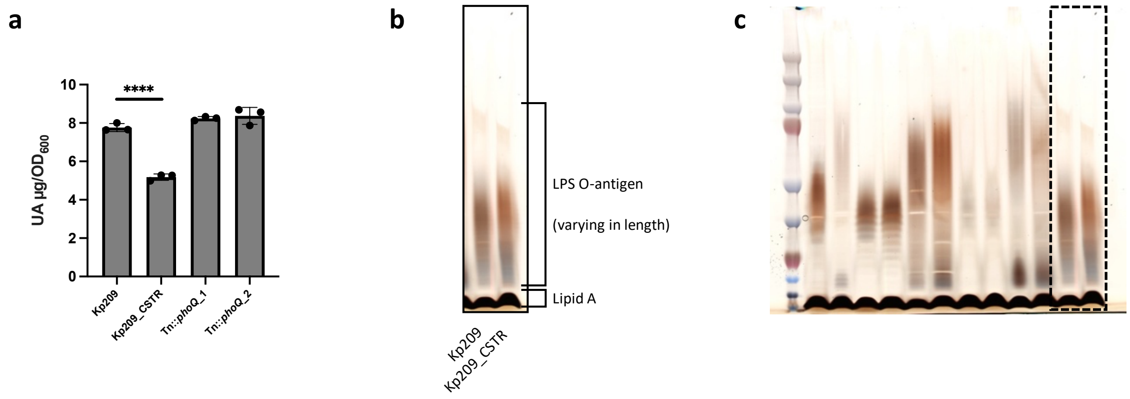

Supplementary figure SI5

(**a&b**) Total IgG (**a**) and IgM (**b**) binding to Kp209 and Kp209_CSTR in normal human serum (NHS). (**c&d**) IgG and IgM binding to Kp209 (**c**) and *E. coli* MG1655 (**d**) in NHS depleted using *E. coli* MG1655, Kp209 or Kp209_CSTR (Δ*E. coli* NHS, ΔKp209 NHS, and ΔKp209_CSTR NHS, respectively). (**e&f**) Inner membrane permeabilization of *E. coli* MG1655 (**e**) and Kp209_CSTR (**f**) in the presence of 1% NHS, Δ*E. coli* NHS, ΔKp209 NHS, or ΔKp209_CSTR NHS (**f**) ΔKp209_CSTR NHS was supplemented with physiological concentrations of IgM (+IgM; 15 µg/ml in 1% NHS) isolated from individual serum donors. (**g**) IgM binding to Kp209 and Kp209_CSTR in IgM isolated from NHS, or isolated IgM depleted using *E. coli* MG1655, Kp209 or Kp209_CSTR (Δ*E. coli* IgM, ΔKp209 IgM, and ΔKp209_CSTR IgM, respectively). (**h**) IgM binding to Kp209 and Kp209_CSTR with IgM isolated from NHS (dotted), IgM depleted with Kp209 (grey) or Kp209_CSTR (orange). Slitted line corresponds to the buffer control. Geometric mean fluorescent intensity (gMFI) and number of events normalized to mode are depicted on the X- and Y-axis, respectively. The graph is representative for three independent repeats. (**i**) IgM biding to *E. coli* MG1655 in IgM isolated from NHS, Δ*E. coli* IgM, ΔKp209 IgM, or ΔKp209_CSTR IgM. (**a-d&g-i**) IgG binding was performed in 0.3% NHS. IgM binding was performed in 10% (depleted) NHS or 45 µg/ml (depleted) IgM isolated from NHS. Binding was detected using anti-hu-IgG-AF647 or anti-hu-IgM-FITC by flow cytometry. Flow cytometry data are represented by gMFI values of bacterial populations. Data represent mean ± standard deviation of three independent experiments. (**e&f)** Bacteria were incubated at 37 °C in the presence of 1 µM SYTOX green nucleic acid stain, and inner membrane permeabilization (SYTOX fluorescence intensity) was detected after (**e**) 60 or (**f**) 90 minutes. Data represents mean ± standard deviation of a minimal of two independent experiments. (**a&b**) Statistical analysis was performed using a nonlinear regression with a variable slope comparing the logEC50 via sum-of squares F test, (**c,d,g&i**) a paired one-way ANOVA with a Tukey's multiple comparisons on Log_{10}-transformed gMFI data, (**e**) or a paired one-way ANOVA with a Tukey's multiple comparisons test on SYTOX fluorescence intensity data.

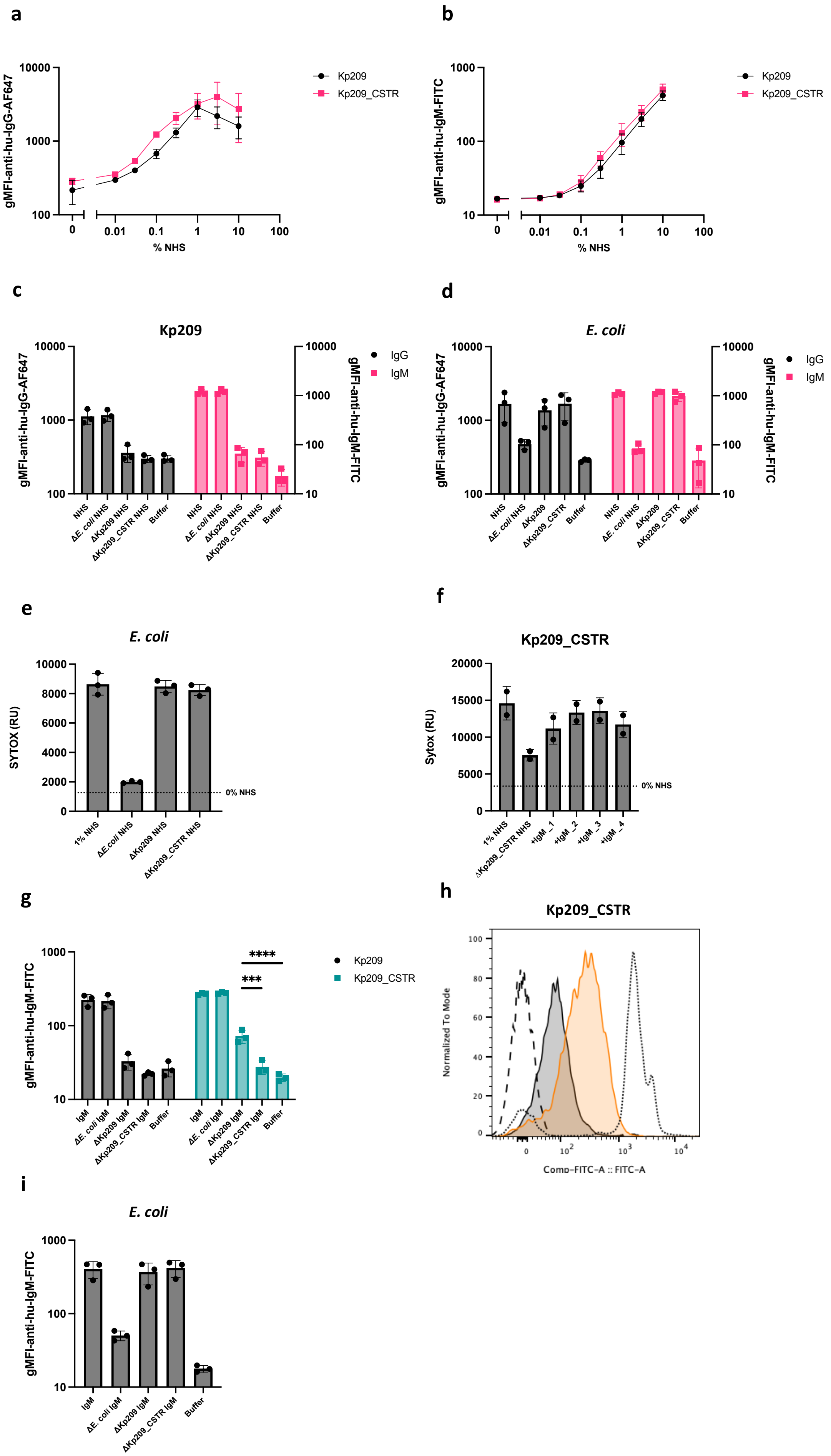
a
gMFI-anti-hu-IgG-AF647
10000
1000
100
0
0.01
0.1
1
10
100
% NHS
Kp209
Kp209_CSTR
b
gMFI-anti-hu-IgM-FITC
1000
100
10
c
Kp209
IgG
IgM
gMFI-anti-hu-IgM-FITC
NHS
ΔE. coli NHS
ΔKp209 NHS
ΔKp209_CSTR NHS
Buffer
d
E. coli
ΔKp209
ΔKp209_CSTR
e
SYTOX (RU)
0% NHS
1% NHS
ΔE.coli NHS
f
Kp209_CSTR
Sytox (RU)
+IgM _1
+IgM _2
+IgM _3
+IgM _4
g
IgM
ΔE. coli IgM
ΔKp209 IgM
ΔKp209_CSTR IgM
****
***
h
Normalized To Mode
Comp-FITC-A :: FITC-A
i

Supplementary table SI1 Differentially expressed genes in Kp209_CSTR compared to Kp209

Kpn209S ID	Name/prediction	$Log_2(FC)$	P_{adj}
**Upregulated genes ($Log_2(FC)>1$, $P_{adj}<0.05$)**			
Kpn209S_01234	*mgtE.1*	5.25	1.06E-195
Kpn209S_03326	*mgtC*	4.15	3.81E-90
Kpn209S_03165	-	3.87	3.69E-57
Kpn209S_03486	*iolT*	3.79	1.12E-45
Kpn209S_01244	*arnC*	3.51	9.15E-146
Kpn209S_03613	-	3.42	1.46E-40
Kpn209S_01243	*arnB*	3.42	5.53E-98
Kpn209S_01954	-	3.39	5.43E-42
Kpn209S_01095	*clcB*	3.38	2.77E-69
Kpn209S_03674	-	3.35	1.09E-39
Kpn209S_03723	*yabI*	3.28	1.08E-51
Kpn209S_00443	*mgtA*	3.24	1.48E-51
Kpn209S_01245	*arnA*	3.22	5.16E-78
Kpn209S_04650	*ctpF*	3.16	2.08E-61
Kpn209S_02782	-	3.15	2.28E-52
Kpn209S_01246	*arnD*	3.11	2.67E-90
Kpn209S_04711	*scrB*	3.04	1.29E-21
Kpn209S_04649	*mdtA*	3.04	7.99E-46
Kpn209S_04702	*btuF*	3.03	2.00E-42
Kpn209S_01247	*arnT*	3.00	8.63E-102
Kpn209S_02647	*pmrD*	3.00	1.73E-56
Kpn209S_03320	*mamB*	2.98	1.40E-25

Kpn209S_04708	*scrK*	2.91	9.08E-19
Kpn209S_03612	-	2.91	2.84E-23
Kpn209S_01631	*ribB*	2.91	1.21E-49
Kpn209S_04552	*pagP*	2.83	3.30E-52
Kpn209S_02224	-	2.81	9.56E-50
Kpn209S_04703	*hmuU*	2.76	1.72E-35
Kpn209S_00310	-	2.75	1.24E-28
Kpn209S_02223	-	2.72	2.16E-52
Kpn209S_02797	-	2.66	2.38E-45
Kpn209S_03487	*fruB*	2.62	1.69E-11
Kpn209S_01249	*arnF*	2.60	2.38E-43
Kpn209S_04337	*ybjG*	2.56	1.08E-51
Kpn209S_04704	*fhuC*	2.53	5.59E-30
Kpn209S_03568	-	2.50	1.38E-15
Kpn209S_02225	-	2.48	2.67E-29
Kpn209S_04648	*bepG*	2.42	3.12E-32
Kpn209S_04492	*kdpA*	2.38	8.00E-22
Kpn209S_02460	*apbE*	2.36	4.22E-44
Kpn209S_04416	*bioB*	2.34	4.79E-28
Kpn209S_04298	*macA*	2.34	1.63E-55
Kpn209S_04087	*phoP*	2.34	1.41E-29
Kpn209S_05121	-	2.34	1.16E-22
Kpn209S_02783	-	2.34	1.54E-13
Kpn209S_03672	*zntB*	2.32	1.10E-19
Kpn209S_02461	*ada*	2.27	1.97E-46

Kpn209S_01787	*fimA*	2.27	6.99E-06
Kpn209S_01248	*arnE*	2.27	9.90E-21
Kpn209S_03301	*maeA*	2.22	1.23E-42
Kpn209S_03166	*slyB*	2.17	2.38E-19
Kpn209S_01782	*fimH*	2.15	2.94E-05
Kpn209S_04297	*macB*	2.12	5.55E-33
Kpn209S_04415	*bioF*	2.11	1.50E-17
Kpn209S_00268	*mdoB*	2.11	1.69E-32
Kpn209S_04710	*sacX*	2.10	3.80E-05
Kpn209S_02462	*alkB*	2.06	9.26E-22
Kpn209S_03599	*speG*	2.06	2.33E-35
Kpn209S_00627	*nhaK*	2.05	5.59E-43
Kpn209S_01786	*fimC*	2.05	8.39E-05
Kpn209S_04088	*phoQ*	2.04	2.00E-24
Kpn209S_01785	*fimD*	2.03	9.59E-05
Kpn209S_04814	*yheI*	1.98	9.73E-24
Kpn209S_04414	*bioC*	1.98	9.80E-15
Kpn209S_01784	*fimF*	1.96	1.91E-04
Kpn209S_01788	*fim*	1.96	1.97E-04
Kpn209S_04709	*scrY*	1.95	2.35E-04
Kpn209S_03142	*mdtK*	1.93	3.93E-16
Kpn209S_02206	*pgpC*	1.89	4.07E-19
Kpn209S_04101	*dmdB*	1.88	2.68E-16
Kpn209S_04100	*betB*	1.87	8.48E-13
Kpn209S_01783	*fimG*	1.86	5.10E-04

Kpn209S_03183	*ydgT*	1.86	2.63E-17
Kpn209S_03598	-	1.85	5.05E-17
Kpn209S_00017	*yaeQ*	1.79	3.31E-27
Kpn209S_01781	-	1.74	1.55E-03
Kpn209S_02799	*ftsI*	1.72	5.67E-09
Kpn209S_04413	*bioD*	1.69	2.68E-08
Kpn209S_04493	*kdpB*	1.66	1.54E-10
Kpn209S_03300	-	1.61	5.59E-06
Kpn209S_04249	*dpaL*	1.60	7.22E-04
Kpn209S_03185	*ydgJ*	1.60	8.07E-16
Kpn209S_03327	*zapE*	1.58	1.39E-14
Kpn209S_00693	-	1.56	5.13E-17
Kpn209S_04813	*mdlB*	1.56	5.91E-14
Kpn209S_01938	*ppnN*	1.54	9.77E-13
Kpn209S_04677		1.52	9.18E-05
Kpn209S_04467	*ybgC*	1.50	3.59E-12
Kpn209S_03728	*ompN*	1.49	9.90E-05
Kpn209S_04757	*fetA*	1.49	3.69E-15
Kpn209S_01476	*mlaF*	1.48	1.74E-16
Kpn209S_03182	*ydgK*	1.47	1.84E-08
Kpn209S_03933	*pfeA*	1.44	6.22E-09
Kpn209S_01523	*osmY*	1.41	1.08E-17
Kpn209S_01538	*gatY*	1.40	8.90E-03
Kpn209S_04466	*tolQ*	1.38	6.13E-11
Kpn209S_01477	*mlaE*	1.36	3.59E-16

Kpn209S_03932	*yciB*	1.33	8.40E-10
Kpn209S_01121	-	1.33	2.00E-07
Kpn209S_04756	*fetB*	1.32	1.22E-12
Kpn209S_01524	*diaA*	1.31	9.16E-15
Kpn209S_02488	*lpxT*	1.31	9.59E-05
Kpn209S_00015	*nlpE*	1.30	6.10E-15
Kpn209S_01790	-	1.29	6.40E-09
Kpn209S_04812	*glnK*	1.25	2.66E-03
Kpn209S_00337	-	1.25	1.87E-08
Kpn209S_01636	*tolC*	1.25	7.20E-11
Kpn209S_04729	*lpxL*	1.24	6.57E-09
Kpn209S_01478	*mlaD*	1.24	6.74E-14
Kpn209S_04846	*yajR*	1.22	1.74E-07
Kpn209S_04464	-	1.20	4.50E-08
Kpn209S_02092	-	1.20	1.52E-05
Kpn209S_01480	*mlaB*	1.19	3.77E-13
Kpn209S_04338	*deoR*	1.19	1.50E-08
Kpn209S_04465	*tolR*	1.18	5.96E-08
Kpn209S_00016	*arfB*	1.18	6.49E-13
Kpn209S_03847	-	1.17	1.63E-02
Kpn209S_04469	*cydX*	1.12	1.16E-02
Kpn209S_00761	*tatA*	1.11	8.31E-11
Kpn209S_03299	-	1.11	4.30E-02
Kpn209S_05125	*umuC*	1.11	1.37E-03
Kpn209S_01479	*mlaC*	1.11	2.31E-12

Kpn209S_04468	-	1.08	7.62E-05
Kpn209S_04470	*cydB*	1.05	1.10E-07
Kpn209S_01122	-	1.04	1.31E-05
Kpn209S_00758	*tatD*	1.04	4.59E-08
Kpn209S_04712	*cra_*	1.03	1.59E-07
**Down regulated genes Log_2(FC)<-1, P_{adj}<0.05)**			
Kpn209S_04410	-	-3.28	2.33E-35
Kpn209S_04407	*eptA*	-2.78	2.52E-23
Kpn209S_02806	*yoaE*	-2.60	7.03E-34
Kpn209S_00354	-	-2.47	7.68E-24
Kpn209S_00355	-	-2.26	1.78E-21
Kpn209S_04408	*pmrA*	-2.16	3.29E-16
Kpn209S_00356	-	-1.97	4.50E-08
Kpn209S_02517	*oprB*	-1.81	1.16E-06
Kpn209S_04409	*pmrB*	-1.80	7.02E-12
Kpn209S_04419	*proY*	-1.72	1.04E-05
Kpn209S_04252	*yedS*	-1.70	1.25E-19
Kpn209S_04420	*hutH*	-1.69	8.22E-05
Kpn209S_03684	*cycA*	-1.67	2.39E-05
Kpn209S_00357	-	-1.66	1.30E-06
Kpn209S_04421	*hutU*	-1.64	2.94E-05
Kpn209S_02966	*rutC*	-1.60	7.18E-04
Kpn209S_02520	*bglB*	-1.59	4.59E-08
Kpn209S_01312	*feoA*	-1.57	8.04E-05
Kpn209S_02514	*yohK*	-1.57	6.19E-05

Kpn209S_00358	*ufaA*	-1.56	9.23E-07
Kpn209S_03046	-	-1.46	3.37E-03
Kpn209S_02515	-	-1.44	5.42E-06
Kpn209S_03019	-	-1.42	2.42E-03
Kpn209S_03686	*astB*	-1.40	1.28E-03
Kpn209S_03685	*astE*	-1.38	4.42E-03
Kpn209S_02519	*bglF*	-1.34	2.49E-08
Kpn209S_01311	*feoB*	-1.33	2.72E-03
Kpn209S_02189	*grcA*	-1.32	4.25E-03
Kpn209S_02830	*dadX*	-1.32	5.04E-05
Kpn209S_00849	*zinT*	-1.30	2.80E-06
Kpn209S_03595	*por*	-1.30	7.66E-03
Kpn209S_04342	-	-1.28	1.74E-02
Kpn209S_03687	*astD*	-1.28	7.21E-03
Kpn209S_03635	*ttdT*	-1.27	2.02E-03
Kpn209S_01194	-	-1.27	1.74E-07
Kpn209S_00616	*acs*	-1.25	7.97E-04
Kpn209S_02967	*dadA*	-1.24	9.09E-03
Kpn209S_02984	-	-1.18	7.67E-03
Kpn209S_00254	-	-1.18	1.80E-02
Kpn209S_01240	*nikA*	-1.16	7.75E-04
Kpn209S_00625	*cidA*	-1.16	9.95E-04
Kpn209S_02511	*mglC*	-1.14	8.95E-03
Kpn209S_02084	*nrdH*	-1.13	9.03E-03
Kpn209S_03634	*namA*	-1.11	2.34E-03

Kpn209S_05120	*scrK*	-1.10	4.96E-04
Kpn209S_04178	*putA*	-1.10	5.78E-04
Kpn209S_02491	*uxuA*	-1.09	5.87E-04
Kpn209S_02422	*nuoG*	-1.08	5.10E-07
Kpn209S_02421	*nuoF*	-1.08	5.66E-05
Kpn209S_05135	-	-1.08	9.26E-06
Kpn209S_01195	*bcsQ*	-1.07	4.63E-04
Kpn209S_00362	-	-1.07	1.05E-02
Kpn209S_05141	-	-1.06	5.51E-03
Kpn209S_01175	*kdgK*	-1.06	3.85E-02
Kpn209S_04476	*sdhB*	-1.06	7.56E-03
Kpn209S_03607	*bioD*	-1.05	9.26E-03
Kpn209S_02450	*glpQ*	-1.05	1.37E-04
Kpn209S_04789	*ykgO*	-1.05	6.90E-03
Kpn209S_03594	*uspG*	-1.01	2.78E-02
Kpn209S_02049	*srlE*	-1.01	1.79E-02

Supplementary table SI2. Real-time PCR primers

Primer	Sequence (5'→ 3')	T_M (°C)	Amplicon size (bp)	Reference
arnD-F	AACTACTGACCATGGCGGCG	61.5	116	12*
arnD-R	GCCAGCCAGTTCACCACGAA	62.5		12*
mgtE-F	TGGTGTGCATTACCCTGTG	57.0	100	This study
mgtE-R	ATAAACGGCGCGGAAACTA	56.3		This study
rpoB-F	GATCCGTGGCGTGACTTATT	55.0	118	54
rpoB-R	GCCCATGTAGACTTCTTGTTCT	54.6		54

*Reference 12 used the alternative gene name *pgbP* for *arnD*
